# Association Between Prognostic Nutritional Index and Disease Activity in Patients with Rheumatoid Arthritis: A Retrospective Cohort Study

**DOI:** 10.31138/mjr.271125.eqa

**Published:** 2026-06-01

**Authors:** Melih Pamukcu, Tugba Izci Duran, Mehmet Akif Baltacı, Mehmet Emin Demir

**Affiliations:** 1 Etlik City Hospital, Health Sciences University, Department of Rheumatology, Ankara, Türkiye;; 2 Denizli Devlet Hastanesi, Denizli, Türkiye;; 3 Ankara Medicana International Hospital, Clinic of Nephrology, Ankara, Türkiye

**Keywords:** prognostic nutritional index, rheumatoid arthritis, DAS28-CRP, HAQ, inflammation

## Abstract

The prognostic nutritional index (PNI), derived from serum albumin and lymphocyte count, reflects immunonutritional status and may be influenced by systemic inflammation. This study examined the association between PNI and disease activity in rheumatoid arthritis (RA). A total of 730 patients fulfilling the 2010 ACR/EULAR criteria were retrospectively analysed. Demographic, clinical, and laboratory data were collected; disease activity and disability were assessed using the DAS28-CRP and Health Assessment Questionnaire (HAQ). Correlation analyses, multivariable linear regression, ROC analyses, subgroup testing, and mediation analyses were performed. The mean age was 56.2±12.5 years and 76.3% were women. Median DAS28-CRP was 3.38, with 11.2% classified as having high disease activity. Mean PNI was 54.0±5.34. PNI was significantly lower in patients with high DAS28-CRP and in severe HAQ categories (p<0.01). PNI correlated negatively with DAS28-CRP, HAQ, CRP, ESR, and disease duration (all p<0.01). However, PNI was not independently associated with DAS28 or HAQ in adjusted regression models, whereas CRP and ESR remained significant predictors. ROC performance of PNI was modest (AUC 0.65–0.67). Associations were stronger in older adults and in men, and mediation analyses indicated that CRP and ESR largely explained the PNI–DAS28 relationship. Lower PNI was associated with greater RA burden, but this association appears to be largely driven by systemic inflammation.

## INTRODUCTION

Rheumatoid arthritis (RA) is a chronic systemic autoimmune disease characterised by persistent synovial inflammation, leading to progressive joint destruction, disability, and impaired quality of life. It affects approximately 0.5–1% of the global population.^1^ Current European League Against Rheumatism (EULAR) and American College of Rheumatology (ACR) treatment guidelines emphasise achieving and maintaining remission or low disease activity using a treat-to-target approach, which requires reliable and objective assessment tools for disease monitoring.^2,3^

Rheumatoid arthritis disease activity is commonly evaluated using composite indices such as the Disease Activity Score 28-C-reactive protein (DAS28-CRP), Clinical Disease Activity Index (CDAI), Simplified Disease Activity Index (SDAI), and patient-reported outcomes such as the Health Assessment Questionnaire (HAQ) and Routine Assessment of Patient Index Data with 3 Measurements (RAPID-3).^4–7^ Although inflammatory markers such as C-reactive protein (CRP) and erythrocyte sedimentation rate (ESR) are incorporated into some of these indices, additional objective biomarkers are needed to better capture the inflammatory and metabolic burden of the disease.^7^

The prognostic nutritional index (PNI), calculated from serum albumin and lymphocyte count, is widely used as an immuno-nutritional marker and has been linked to outcomes in cancer and several autoimmune diseases.^8–13^ Inflammation-associated hypoalbuminaemia and lymphopenia are common in RA, suggesting that PNI may reflect both nutritional status and inflammatory activity.^11–14^ However, despite increasing interest, large-scale data on the relationship between PNI and RA disease activity remain limited. This study therefore aimed to evaluate the association between PNI, disease activity (DAS28-CRP), and functional disability (HAQ) in patients with RA.

## METHODS

This study was designed and reported in accordance with the Strengthening the Reporting of Observational Studies in Epidemiology (STROBE) guidelines.^15^

### Study Population

This retrospective observational study included consecutive adult patients diagnosed with RA according to the 2010 ACR/EULAR classification criteria^16^ who followed between January 1, 2018, and June 1, 2021. Consecutive patients meeting the inclusion criteria were enrolled to minimise selection bias.

Patients aged ≥18 years who had been followed in the rheumatology clinic for at least three months were eligible. Of 773 screened patients, 43 were excluded based on predefined criteria (cancer, heart failure, liver or kidney disease, haematological disorders, pregnancy, acute or chronic infections, other rheumatic diseases, or those using >7.5 mg of prednisolone daily, or those with conditions that could affect albumin or lymphocyte levels), leaving 730 patients for the final analysis. A total of 730 patients were included in the study. The mean age of the study population was 56.2 ± 12.5 years, and 557 patients (76.3%) were female. The median disease duration was 51.7 months. Baseline demographic and clinical characteristics of the study population are summarised in **Table 1**.

**Table 1.** Baseline demographic, clinical, and laboratory characteristics.

**Parameters**	**All patients (n=730)**
Age (years), mean±SD	56.2±12.5
Female sex, n (%)	557 (76.3)
Disease duration (months), mean±SD	51.7±12.5
**Disease activity scores**	
**DAS28-CRP, median (IQR)**	3.38 (1.68)
Remission, n (%)	159 (21.8)
Low activity, n (%)	109 (14.9)
Moderate activity, n (%)	380 (52.1)
High activity, n (%)	82 (11.2)
**HAQ**	0.69±0.41
Mild to moderate, n (%)	579 (79.3)
Moderate to severe, n (%)	138 (18.9)
Severe to very severe, n (%)	12 (1.6)
**Laboratory results**	
Haemoglobin (g/dL), mean±SD	13.1±1.55
Albumin (g/dL), median (IQR)	4.34 (0.41)
Creatinine (mg/dL), median (IQR)	0.72 (0.23)
CRP (mg/L), median (IQR)	6.13 (11.1)
ESR (mm/h), median (IQR)	16 (16)
Lymphocytes, (μ/L), median (IQR)	2040 (978)
PNI, mean±SD	54±5.34
RF positivity, n (%)	482 (66)
Anti-CCP positivity, n (%)	372 (51)
RF and/or Anti-CCP positivity, n (%)	528 (72.3)

CCP: Cyclic citrullinated peptide; CRP: C-reactive protein; DAS28-CRP: 28-joint Disease Activity Score-C reactive protein; ESR: Erythrocyte sedimentation rate; HAQ: Health Assessment Questionnaire; PNI: Prognostic nutritional index; RF: Rheumatoid factor.

### Data collection and Definitions

Demographic characteristics, disease duration, medication use, comorbidities, and lifestyle factors (such as smoking and alcohol use) were recorded. Laboratory evaluations included CRP, ESR, creatinine, serum albumin, rheumatoid factor (RF), and anti-cyclic citrullinated peptide (anti-CCP). Complete blood counts were used to obtain leukocyte differentials. The PNI was calculated using the formula: 10 × serum albumin (g/dL) + 0.005 × lymphocyte count (/mm^3^).

All patients underwent systemic and rheumatologic physical examinations. Disease activity was assessed using the DAS28-CRP ^4^ and functional disability was evaluated using the HAQ.^6^ Both DAS28-CRP and HAQ scores were calculated by a rheumatologist. All measurement tools used in the study have validated Turkish versions.

### Measurement tools

#### Disease Activity Score 28-joint count C-reactive protein

Disease Activity Score 28-joint count C-reactive protein is a composite index used to assess rheumatoid arthritis disease activity based on the number of tender and swollen joints, CRP level, and the patient’s global assessment ^4^. Scores range from 0 to 9.4, with <2.6 indicating remission, 2.6–3.2 low disease activity, 3.2–5.1 moderate disease activity, and >5.1 high disease activity.

#### Health Assessment Questionnaire

The HAQ is a validated patient-reported measure of functional status in rheumatoid arthritis ^6^. It evaluates difficulty in performing daily activities across eight domains (dressing, arising, eating, walking, hygiene, reaching, gripping, and usual activities). Each item is scored from 0 to 3, where 0 indicates no difficulty and 3 indicates inability to perform the activity. The final HAQ score is calculated as the mean of all items and ranges from 0 to 3, with higher scores reflecting greater functional disability. The Turkish-adapted version of the instrument has been validated and demonstrated good reliability, and it is widely used in clinical and epidemiological research.^17^

Potential sources of bias were considered. Selection bias was minimised by including consecutive patients. Patients with conditions known to influence albumin or lymphocyte levels were excluded to reduce measurement bias. Residual confounding cannot be fully excluded due to the observational design.

### Statistical analysis

All analyses were performed using SPSS version 23.0 (SPSS Inc., Chicago, IL, USA). Categorical variables were expressed as numbers and percentages, and numerical variables as mean ± standard deviation or median (IQR), depending on distribution. Normality was assessed using the Kolmogorov–Smirnov test.

Between-group comparisons were conducted using the independent samples *t* test or Mann–Whitney U test for continuous variables, and categorical variables were compared using the chi-square test. Associations between the PNI and clinical or laboratory parameters were evaluated using Spearman correlation analysis. Multivariable linear regression models were constructed to examine the associations between PNI and disease activity (DAS28-CRP) as well as functional disability (HAQ), while controlling for potential confounders. Covariates included age, sex, disease duration, serological status (RF and anti-CCP positivity), inflammatory markers (CRP and ESR), and medication use.

Receiver operating characteristic (ROC) analyses assessed the discriminatory ability of PNI for high DAS28-CRP (>5.1) and severe HAQ (>2.0). Optimal cutoff values were determined using the Youden index. Subgroup analyses (age groups and sex), interaction terms (PNI×CRP, PNI×ESR), propensity-score matching (1:1 nearest neighbour), and sensitivity analyses using winsorising (1st–99th percentiles) were also performed.

There were no missing data for the primary variables included in the analysis; therefore, no imputation methods were required. A two-sided *p* value < 0.05 was considered statistically significant.

## RESULTS

RF and/or anti-CCP positivity was present in 72.3%. The median DAS28-CRP was 3.38, and 11.2% of patients had high disease activity (>5.1). The mean PNI was 54.0±5.34. Baseline demographic and laboratory characteristics are presented in **Table 1**. The distribution of PNI values is presented in **Supplementary Figure S1**. Patients with high DAS28-CRP exhibited markedly reduced PNI levels (51.52±5.73) compared with those in remission or with low and moderate activity (54.30±5.45, 54.25±5.64, and 54.00±6.46; p<0.001). PNI also declined progressively across HAQ categories, reaching the lowest values in patients with severe-to-very severe disability (p=0.003). PNI values differed significantly across DAS28-CRP and HAQ categories (p<0.01; **Supplementary Figure S2**).

Correlation analyses between PNI and clinical/laboratory parameters are summarised in **Table 2**. PNI showed significant negative correlations with DAS28-CRP, HAQ scores, ESR, and CRP (all p<0.005). Scatter-plots illustrating these relationships are provided in **Supplementary Figure S3**.

**Table 2.** Spearman correlations between PNI and clinical/laboratory variables.

	**Rho value**	**P value**
**DAS28-CRP**	−0.127	0.001
**HAQ**	−0.093	0.012
**CRP**	−0.123	0.001
**ESR**	−0.182	<0.001
**Disease duration**	−0.168	<0.001

CRP: C-reactive protein; DAS28-CRP: 28-joint Disease Activity Score- C-reactive protein; ESR: Erythrocyte sedimentation rate; HAQ: Health Assessment Questionnaire.

In multivariable linear regression analyses, PNI was not independently associated with either disease activity or functional disability after adjustment for confounders. In the DAS28-CRP model, PNI showed no significant association (β=–0.0011, 95% CI –0.0129 to 0.0107; p=0.855). CRP (β=0.0462, p<0.001) and ESR (β=0.0139, p<0.001) emerged as the strongest independent predictors of disease activity, while medication use also remained significant (β=0.0837, p=0.024). Age, sex, disease duration, RF, and anti-CCP positivity were not associated with DAS28-CRP.

Similarly, in the HAQ model, PNI again demonstrated no independent effect (β=0.0022, 95% CI –0.0022 to 0.0066; p=0.333). As with disease activity, CRP (β=0.0175, p<0.001) and ESR (β=0.0048, p<0.001) remained significant determinants of functional disability, whereas age, sex, disease duration, RF, anti-CCP, and medication use were not significant contributors (**Table 3**).

**Table 3.** Multivariable linear regression: determinants of DAS28-CRP and HAQ.

**Outcome**	**Variable**	**β**	**Standard Error**	**t**	**p**	**95% CI Lower**	**95% CI Upper**
**DAS28**	PNI	−0.0011	0.006	−0.18	0.855	−0.0129	0.0107
	Age	0.011	0.0063	1.76	0.079	−0.0013	0.0233
	Gender	−0.1104	0.0775	−1.42	0.155	−0.2626	0.0418
	Disease Duration	−0.0049	0.0063	−0.78	0.435	−0.0172	0.0074
	CRP	0.0462	0.0027	17.14	0.0	0.0409	0.0515
	ESR	0.0139	0.0028	5.03	0.0	0.0085	0.0193
	RF positivity	0.0107	0.0734	0.15	0.884	−0.1335	0.1549
	CCP positivity	0.0077	0.071	0.11	0.913	−0.1317	0.1471
	Medication	0.0837	0.0369	2.27	0.024	0.0112	0.1562
**HAQ**	PNI	0.0022	0.0022	0.97	0.333	−0.0022	0.0066
	Age	0.0035	0.0023	1.49	0.137	−0.0011	0.0081
	Gender	−0.0365	0.0289	−1.26	0.208	−0.0932	0.0203
	Disease Duration	0.0008	0.0023	0.35	0.73	−0.0038	0.0054
	CRP	0.0175	0.001	17.38	0.0	0.0155	0.0194
	ESR	0.0048	0.001	4.62	0.0	0.0027	0.0068
	RF positivity	0.0161	0.0274	0.59	0.558	−0.0377	0.0699
	CCP positivity	0.0174	0.0265	0.66	0.512	−0.0346	0.0694
	Medication	0.0168	0.0138	1.22	0.223	−0.0103	0.0438

ROC analysis showed that PNI had modest ability to discriminate clinically significant outcomes. For high DAS28-CRP (>5.1), the AUC was 0.65 with an optimal cut-off of 51.15 (48% sensitivity, 76% specificity). For severe disability (HAQ >2.0), the AUC was 0.67 with a cut-off of 53.80 (83% sensitivity, 50% specificity). Detailed ROC metrics are presented in **Table 4**.

**Table 4.** ROC performance of PNI for high disease activity and severe functional disability.

**Outcome**	**AUC**	**Cut-off (Youden)**	**Sensitivity (%)**	**Specificity (%)**
High DAS28 (>5.1)	0.65	51.15	48	76
Severe HAQ (>2.0)	0.67	53.80	83	50

Subgroup analyses demonstrated pronounced ageand sex-related differences in the association between PNI and disease outcomes. Among elderly patients (>65 years), PNI values were significantly lower (mean 52.6 ± 6.0), and significant negative correlations were observed with both DAS28 (r = –0.261, p = 0.001) and HAQ (r = –0.238, p = 0.002). These associations were not evident in patients younger than 65 years (**Supplementary Figure S5**).

Gender-stratified analyses showed that men exhibited stronger relationships between PNI and disease outcomes, with significant negative correlations for both DAS28 (r = –0.280, p<0.001) and HAQ (r = –0.286, p<0.001). In women, the correlations were weaker and did not reach statistical significance.

Subgroup ROC analyses further supported these findings, indicating higher discriminatory performance of PNI in elderly patients and in men, whereas discriminatory ability was lower in younger individuals and in women (**Supplementary Table S2**).

Overall, these results suggest that the association between PNI and disease burden may be more pronounced in older adults and males, while its association with disease activity and functional impairment appears attenuated in younger patients and in women. Interaction analyses were performed to determine whether inflammatory markers or disease duration modified the association between PNI and clinical outcomes. For DAS28, a significant interaction was detected between PNI and CRP (β = 0.0017, p = 0.0002), indicating that the relationship between nutritional status and disease activity depended on the level of systemic inflammation. No significant interactions were found with ESR or disease duration.

For HAQ, significant interactions were also observed for PNI × CRP (β = 0.0006, p = 0.0003) and PNI × ESR (β = –0.0005, p = 0.018), suggesting that the effect of PNI on functional disability varied according to inflammatory burden. The interaction between PNI and disease duration was not significant (**Supplementary Figure S6**). Taken together, these findings indicate that the predictive value of PNI is context-dependent and is particularly influenced by inflammatory activity rather than representing a uniform predictor across all patient groups. This pattern aligns with the significant PNI × CRP interaction observed in the regression analyses, indicating that the influence of nutritional status on disease activity varies according to the level of systemic inflammation.

After propensity-score matching (1:1 nearest neighbour) for age, sex, and disease duration, 26 matched patient pairs were identified. Patients with low PNI (<45) demonstrated higher DAS28-CRP scores and worse HAQ outcomes compared with those with high PNI (≥45), confirming the association between reduced nutritional status and increased disease burden (**Supplementary Table S2**).

Mediation analyses showed that the relationship between PNI and DAS28-CRP was largely explained by systemic inflammation. CRP demonstrated a significant indirect effect (estimate –0.0201, 95% CI –0.030 to –0.0106), accounting for approximately 84% of the total effect, whereas the direct effect was non-significant. ESR also showed a significant indirect effect (estimate –0.0122, 95% CI –0.0183 to –0.0064), accounting for roughly 51% mediation. These findings indicate that CRP and ESR partially explained the association between PNI and RA disease activity.

To assess robustness, winsorising (1st–99th percentile) was applied to PNI, CRP, ESR, DAS28-CRP, and HAQ. The association between PNI and both outcomes remained stable. For DAS28-CRP, the β coefficient for PNI changed minimally from –0.024 (p=0.0016) to –0.025 (p=0.0019); for HAQ, β changed from –0.0066 (p=0.0196) to –0.0069 (p=0.0204). These results confirm that the observed associations were not driven by outliers.

## DISCUSSION

The PNI is an easily calculated measure reflecting both nutritional and immunological status and may therefore be relevant in RA. In this retrospective observational study, lower PNI values were associated with higher disease activity and worse functional status; however, these associations were modest. Importantly, PNI did not remain independently associated with disease activity or disability after adjustment for relevant clinical and inflammatory covariates, indicating that the observed relationships are largely driven by systemic inflammation. Accordingly, correlations between PNI and DAS28-CRP or HAQ scores should be interpreted as reflecting inflammatory burden rather than an independent effect of PNI. Previous studies, primarily in oncology, have validated the prognostic utility of PNI for predicting postoperative complications and mortality,^22–24^ and accumulating evidence suggests that its usefulness extends to chronic inflammatory diseases as well.^25,26^

Our findings are broadly consistent with earlier RA-focused studies reporting inverse associations between PNI and disease activity measures. Öz et al. reported a negative correlation between PNI and DAS28 in a smaller RA cohort,^25^ while analyses from the NHANES database demonstrated associations between higher PNI levels and lower RA prevalence and mortality.^26^ Other cross-sectional studies have similarly shown lower PNI values in RA patients compared with healthy controls and inverse relationships with disease activity indices.^27^ While optimal PNI cut-off values vary slightly among studies, most reports—including ours—identify thresholds in the range of 40–45 for identifying patients with higher disease activity.

Mechanistically, the association between PNI and RA activity is biologically plausible. Albumin is a negative acute-phase reactant downregulated by IL-6, TNF-α, and IL-1β during active inflammation.^28–30^ Lymphopenia, the second component of PNI, can result from cytokine-driven redistribution of lymphocytes to inflamed tissues, increased apoptosis, or treatment-related effects.^31,32^ Meta-analytic data confirm that RA patients with active disease exhibit lower lymphocyte counts and higher neutrophil and platelet counts.^33^ Thus, a lower PNI may reflect a combined effect of elevated inflammatory activity and declining nutritional or immune reserves.

Importantly, although PNI showed significant unadjusted associations with disease activity and functional disability, it did not remain independently associated with DAS28-CRP or HAQ after adjustment for key confounders, particularly inflammatory markers. CRP and ESR emerged as the dominant determinants of both outcomes, indicating that the observed relationships between PNI and rheumatoid arthritis severity are largely driven by systemic inflammation rather than representing independent effects. Consistent with this interpretation, interaction analyses demonstrated that the association between PNI and clinical outcomes varied according to inflammatory burden, particularly at higher CRP levels. In line with these findings, ROC analyses showed limited to modest discriminatory ability of PNI for identifying high disease activity and severe functional disability, supporting its role as an adjunct marker rather than a standalone clinical tool. Subgroup analyses further indicated that the association between PNI and disease outcomes may be more pronounced in older adults and in male patients, whereas no statistically significant associations were observed in younger individuals or in women. Similar age-dependent patterns have been reported previously, particularly regarding infection risk in elderly RA populations.^34^ These findings may reflect age-related reductions in physiological and immunological reserve, rendering nutritional and inflammatory alterations more clinically relevant in these subgroups. However, given the exploratory nature of these analyses, such findings should be interpreted with caution. The potential clinical implications of our findings merit consideration. First, PNI may serve as an adjunct assessment tool in routine practice because it is calculated from universally available laboratory values. Markedly low PNI values could alert clinicians to poorly controlled inflammation or unrecognised nutritional deficits. This may be especially relevant in resource-limited settings or during telemedicine encounters where full composite scores cannot be obtained. Second, low PNI identifies patients at higher risk of complications such as infections and treatment intolerance.^34^ Thus, patients with low PNI may benefit from targeted nutritional interventions, dietary counselling, or closer monitoring. Observational studies suggest that addressing malnutrition may improve metabolic risk factors and overall disease management.^35^ Whether structured nutritional interventions can improve disease control or modify clinical outcomes in RA requires prospective evaluation. In accordance with STROBE recommendations,^15^ several limitations should be acknowledged. First, the retrospective and cross-sectional design precludes causal inference, and the directionality of the observed associations cannot be determined. Second, although major confounders affecting albumin and lymphocyte counts were excluded, residual confounding cannot be fully ruled out. Third, this was a single-centre study, potentially limiting generalisability to more diverse populations. Nutritional baselines vary across countries and socioeconomic contexts, and cut-off values may differ accordingly. Finally, PNI is a simplified index and does not capture broader aspects of nutritional status such as body composition, sarcopenia, or micronutrient deficiencies. Therefore, the generalisability of these findings may be limited to patients with rheumatoid arthritis followed in similar tertiary-care settings. Moreover, it should be acknowledged that CRP is a component of the DAS28-CRP score by definition; therefore, its strong association with DAS28-CRP is expected. Accordingly, analyses involving CRP and ESR should be interpreted as a decomposition of the inflammatory contribution to the PNI–DAS28 relationship rather than as evidence of causal mediation. Future research should include longitudinal studies to determine whether PNI predicts long-term outcomes such as flares, radiographic progression, disability, and mortality. Intervention studies examining whether improving PNI through nutritional support influences disease activity would also be valuable. Integration of PNI with other inflammatory and nutritional biomarkers may yield more comprehensive prognostic tools tailored for RA.

## CONCLUSION

In conclusion, lower PNI values are associated with higher disease activity and worse functional status in RA; however, these relationships appear to be largely driven by systemic inflammation. While PNI does not independently predict disease activity after adjustment, it may represent a pragmatic adjunct marker, particularly in older patients or contexts where comprehensive assessment tools are unavailable.

## Data Availability

The datasets used and/or analysed during the current study are available from the corresponding author on reasonable request.

## USE OF ARTIFICIAL INTELLIGENCE

Artificial intelligence (AI) tools, specifically ChatGPT (OpenAI, San Francisco, CA, USA), were employed to support the preparation of this manuscript. AI was used to assist in language refinement, improving clarity, grammar, and flow, and to help structure certain sections of the discussion and conclusion. All scientific content, data interpretation, and final conclusions are the responsibility of the authors. The authors carefully reviewed and verified all AI-assisted text to ensure accuracy and integrity. No AI tools were used to generate, analyse, or manipulate study data.

## References

[1] Pamukcu M İzci Duran T Ulusoy H Altınbaş K. Investigation of the correlation between mood disorder symptoms and disease activity and functional status in rheumatoid arthritis patients. Turk J Med Sci 2021;51:3008–16. DOI: 10.3906/sag-2107-283. 34773692 PMC10734842

[2] Smolen JS Landewé RBM Bergstra SA Kerschbaumer A Sepriano A Aletaha D EULAR recommendations for the management of rheumatoid arthritis with synthetic and biological disease-modifying antirheumatic drugs: 2022 update. Ann Rheum Dis 2023;82:3–18. DOI: 10.1136/ard-2022-223356. 36357155

[3] Fraenkel L Bathon JM England BR St Clair EW Arayssi T Carandang K 2021 American College of Rheumatology Guideline for the Treatment of Rheumatoid Arthritis. Arthritis Care Res (Hoboken) 2021 Jul;73(7):924–39. DOI: 10.1002/acr.24596. 34101387 PMC9273041

[4] van Riel PL. The development of the disease activity score (DAS) and the disease activity score using 28 joint counts (DAS28). Clin Exp Rheumatol 2014;32:S-65-74. 25365092

[5] Aletaha D Smolen J. The Simplified Disease Activity Index (SDAI) and the Clinical Disease Activity Index (CDAI): a review of their usefulness and validity in rheumatoid arthritis. Clin Exp Rheumatol 2005;23:S100–108. 16273793

[6] Maska L Anderson J Michaud K. Measures of functional status and quality of life in rheumatoid arthritis: Health Assessment Questionnaire Disability Index (HAQ), Modified Health Assessment Questionnaire (MHAQ), Multidimensional Health Assessment Questionnaire (MDHAQ), Health Assessment Questionnaire II (HAQ-II), Improved Health Assessment Questionnaire (Improved HAQ), and Rheumatoid Arthritis Quality of Life (RAQoL). Arthritis Care Res 2011;63 Suppl 11:S4–13. DOI: 10.1002/acr.20620. 22588760

[7] Yazici Y Bergman M Pincus T. Time to score quantitative rheumatoid arthritis measures: 28-Joint Count, Disease Activity Score, Health Assessment Questionnaire (HAQ), Multidimensional HAQ (MDHAQ), and Routine Assessment of Patient Index Data (RAPID) scores. J Rheumatol 2008;35:603–9. 18322993

[8] Ishiguro T Aoyama T Ju M Kazama K Fukuda M Kanai H Prognostic Nutritional Index as a Predictor of Prognosis in Postoperative Patients With Gastric Cancer. In Vivo 2023;37:1290–6. DOI: 10.21873/invivo.13207. 37103068 PMC10188044

[9] Tan X Chen H. The Prognostic Value of Prognostic Nutritional Index in Patients with Ovarian Cancer: A Systematic Review and Meta-Analysis. Nutr Cancer 2023;75:73–81. DOI: 10.1080/01635581.2022.2104879. 35900054

[10] Tobing E Tansol C Tania C Sihombing AT. Prognostic Nutritional Index (PNI) as Independent Predictor of Poor Survival in Prostate Cancer: A Systematic Review and Meta-Analysis. Clin Genitourin Cancer 2024;22:102142. DOI: 10.1016/j.clgc.2024.102142. 39079465

[11] Carli L Tani C Vagnani S Signorini V Mosca M. Leukopenia, lymphopenia, and neutropenia in systemic lupus erythematosus: Prevalence and clinical impact--A systematic literature review. Semin Arthritis Rheum 2015;45:190–4. 20150521. DOI: 10.1016/j.semarthrit.2015.05.009. 26170228

[12] Klein A and Molad Y. Hematological Manifestations among Patients with Rheumatic Diseases. Acta Haematol 2021;144: 403–12. DOI: 10.1159/000511759. 33221805

[13] Fessler J Fasching P Raicht A Hammerl S Weber J Lackner A Lymphopenia in primary Sjögren’s syndrome is associated with premature aging of naïve CD4+ T cells. Rheumatology (Oxford, England) 2021;60:588–97. DOI: 10.1093/rheumatology/keaa105. 32227243

[14] Subesinghe S Kleymann A Rutherford AI Bechman K Norton S Benjamin Galloway J. The association between lymphopenia and serious infection risk in rheumatoid arthritis. Rheumatology (Oxford, England) 2020;59:762–6. DOI: 10.1093/rheumatology/kez349. 31504905

[15] von Elm E Altman DG Egger M Pocock SJ Gøtzsche PC Vandenbroucke JP STROBE Initiative. The Strengthening the Reporting of Observational Studies in Epidemiology (STROBE) Statement: Guidelines for Reporting Observational Studies. Ann Intern Med 2007 Oct 16;147(8):573–7. DOI: 10.7326/0003-4819-147-8-200710160-00010. 17938396

[16] Aletaha D Neogi T Silman AJ Funovits J Felson DT Bingham CO 3rd 2010 Rheumatoid arthritis classification criteria: an American College of Rheumatology/European League Against Rheumatism collaborative initiative. Arthritis Rheum 2010 Sep;62(9):2569–81. DOI: 10.1002/art.27584. 20872595

[17] Küçükdeveci AA Sahin H Ataman S Griffiths B Tennant A. Issues in cross-cultural validity: example from the adaptation, reliability, and validity testing of a Turkish version of the Stanford Health Assessment Questionnaire. Arthritis Rheum 2004 Feb 15;51(1):14–9. DOI: 10.1002/art.20091. 14872450

[18] Jin H Wang G Lu Q Rawlins J Chen J Kashyap S Pathophysiology of Myopenia in rheumatoid arthritis. Bone Res 2025;13:64. DOI: 10.1038/s41413-025-00438-9. 40523898 PMC12170877

[19] Cano-García L Redondo-Rodríguez R Manrique-Arija S Domínguez-Quesada C Crisóstomo Vacas J Armenteros-Ortiz P Prevalence of Malnutrition and Associated Factors in Older Patients with Rheumatoid Arthritis: A Cross-Sectional Study. Nutrients 2023;Aug 8;15(16):3500. DOI: 10.3390/nu15163500. 37630691 PMC10460011

[20] Tian P Xiong J Wu W Shi S Chen A Chen K Impact of the malnutrition on mortality in Rheumatoid arthritis patients: A cohort study from NHANES 1999–2014. Front Nutr 2022;9:993061. DOI: 10.3389/fnut.2022.993061. 36687731 PMC9845564

[21] Masuko K. Rheumatoid cachexia revisited: a metabolic comorbidity in rheumatoid arthritis. Front Nutr 2014;1:20. DOI: 10.3389/fnut.2014.00020. 25988122 PMC4428367

[22] Jiang N Deng JY Ding XW Ke B Liu N Zhang RP Prognostic nutritional index predicts postoperative complications and long-term outcomes of gastric cancer. World J Gastroenterol 2014 Aug 14;20(30):10537–44. DOI: 10.3748/wjg.v20.i30.10537. 25132773 PMC4130864

[23] Kim YJ Park HP Kim HS Park S. Preoperative Prognostic Nutritional Index Is a Prognostic Indicator of Cancer-Specific Survival in Patients Undergoing Endometrial Cancer Surgery. J Korean Med Sci 2023 May 29;38(21):e163. DOI: 10.3346/jkms.2023.38.e163. 37270918 PMC10226847

[24] Xu F Meng C Yang Z Li H Gao J Sun L Zhang X Prognostic nutrition index predicts short-term surgical complications in patients with rectal cancer after laparoscopic surgery. Front Surg 2022;9:1000108. DOI: 10.3389/fsurg.2022.1000108. 36386497 PMC9640637

[25] Öz N Gezer HH Cilli Hayıroğlu S Duruöz MT. Evaluation of the prognostic nutritional index (PNI) as a tool for assessing disease activity in rheumatoid arthritis patients. Clin Rheumatol 2024;43:1461–7. DOI: 10.1007/s10067-024-06927-2. 38466500

[26] Wang J Zhu R Fang H Xing X Ge L Cai G. Association of prognostic nutritional index with the presence and all-cause mortality of rheumatoid arthritis: the National Health and Nutrition Examination Survey 2003–2018. BMC Public Health 2024;24:3281. DOI: 10.1186/s12889-024-20795-0. 39593001 PMC11590335

[27] Kılıç Ö Tecer D Canbaş M Kaya MN Çınar M Yılmaz S. Immune nutrition indices are associated with disease activity in rheumatoid arthritis: a cross-sectional study. Biomark Med 2024;18:1093–102. DOI: 10.1080/17520363.2024.2430942. 39573913 PMC11654804

[28] Jain S Gautam V Naseem S. Acute-phase proteins: As diagnostic tool. J Pharm Bioallied Sci 2011;3:118–27. DOI: 10.4103/0975-7406.76489. 21430962 PMC3053509

[29] Bode JG Albrecht U Häussinger D Heinrich PC Schaper F. Hepatic acute phase proteins--regulation by IL-6- and IL-1-type cytokines involving STAT3 and its crosstalk with NF-κB-dependent signaling. Eur J Cell Biol 2012;91:496–505. DOI: 10.1016/j.ejcb.2011.09.008. 22093287

[30] Roy S Kumar V Kumar V Behera BK. Acute Phase Proteins and their Potential Role as an Indicator for Fish Health and in Diagnosis of Fish Diseases. Protein Pept Lett 2017;24:78–89. DOI: 10.2174/0929866524666161121142221. 27903234

[31] Kondo N Kuroda T Kobayashi D. Cytokine Networks in the Pathogenesis of Rheumatoid Arthritis. Int J Mol Sci 2021;10;22(20):10922. DOI: 10.3390/ijms222010922. 34681582 PMC8539723

[32] Ding Q Hu W Wang R Yang Q Zhu M Li M Signaling pathways in rheumatoid arthritis: implications for targeted therapy. Signal Transduct Target Ther 2023;8:68. DOI: 10.1038/s41392-023-01331-9. 36797236 PMC9935929

[33] Mangoni AA Zinellu A. Diagnostic accuracy of the neutrophilto-lymphocyte ratio and the platelet-to-lymphocyte ratio in rheumatoid arthritis: a systematic review and meta-analysis. Clin Exp Med 2024;24:207. DOI: 10.1007/s10238-024-01478-x. 39230596 PMC11374877

[34] Isoda K Tsuji S Harada Y Yoshimura M Nakabayashi A Sato M Potential of the prognostic nutritional index to determine the risk factor for severe infection in elderly patients with rheumatoid arthritis. Mod Rheumatol 2023;33:88–95. DOI: 10.1093/mr/roac001. 35134981

[35] Olsen MN Halse AK Skeie E Lein RK Nilsen RM Tangvik RJ. Effect of dietary interventions on nutritional status in patients with rheumatoid arthritis and spondyloarthritis - A systematic review and meta-analysis. Clin Nutr 2024;43:926–35. DOI: 10.1016/j.clnu.2024.02.019. 38401228

